# Intertidal habitat complexity influences the density of the non-native crab *Hemigrapsus sanguineus*

**DOI:** 10.7717/peerj.15161

**Published:** 2023-04-06

**Authors:** Zachary W. Towne, Michael L. Judge, Nancy J. O’Connor

**Affiliations:** 1 Department of Biology, University of Massachusetts at Dartmouth, Dartmouth, Massachusetts, United States; 2 Department of Biology, Manhattan College, Riverdale, New York, United States

**Keywords:** Asian shore crab, Behavior, Density, *Hemigrapsus sanguineus*, Habitat complexity, Rocky intertidal, Electivity, Bioinvasion

## Abstract

Habitat structural complexity can provide protection from predators, potentially affecting population density of native and non-native prey. The invasive Asian shore crab, *Hemigrapsus sanguineus*, occurs in variable densities in the rocky intertidal zone of eastern North America and northern Europe, often in densities greater than in its native range. The present study examined the influence of habitat complexity on the density of *H. sanguineus*. Artificial shelters of concrete pavers with stones arranged in increasing complexity were deployed in the intertidal zone along a rocky shore in southeastern Massachusetts, USA, for 21 consecutive weekly intervals in 2020. Crabs consistently reached the highest densities in the most complex shelters despite their lower internal surface area. In addition, crabs exhibited shelter selectivity based on body size, with large crabs occupying artificial shelters in greater numbers than adjacent natural substrate. In a subsequent lab study, crab activity over 1 h was observed in the presence of the same artificial shelters, under simulated tidal conditions. Shelter complexity had little influence on the number of crabs under the pavers although crabs were more active when submerged in water than exposed to air. These results show that crab density increases as habitat complexity increases, and complexity may serve as a predictor of *H. sanguineus* density but not short-term behavior.

## Introduction

The introduction of non-native species to new geographic areas continues to occur worldwide (Ruiz et al., 2000; Seebens et al., 2017; Ojaveer et al., 2018). Some non-native species possess traits that contribute to invasion success and population growth, which can lead to impacts on native species (Simberloff et al., 2013; Chan & Briski, 2017). However, densities of invaders can vary within invaded regions for reasons that are not well understood. Many biological factors can affect population density, such as predation by and competition with native species as well as larval supply and recruitment. Habitat characteristics can also be important. For example, substrate composition could affect population densities of benthic species if it provides a refuge from predation as well as appropriate habitable space (Kovalenko, Thomaz & Warfe, 2012).

The effectiveness of a habitat as a refuge is related to its three-dimensional structure. Habitat structure includes both the abundance and variety of abiotic and biotic structural components (McCoy & Bell, 1991). Habitat complexity can also be described in terms of the size, abundance, diversity, scale, and arrangement of physical elements in space (Tokeshi & Arakaki, 2012). The spatial arrangement of structural elements in the marine environment can influence the quality of a habitat as a refuge from predators (Eggleston et al., 1990; Grabowski, 2004; Humphries, La Peyre & Decossas, 2011; Hesterberg et al., 2017; Longmire et al., 2021). For example, macroalgal species with complex structure support greater densities of epifauna (Parker, Duffy & Orth, 2001) and fishes (Srednick & Steele, 2022). In the rocky intertidal zone, the quality of the habitat as a refuge may vary depending on how the structural elements (rocks) are arranged and the amount of crevice space available between rocks. The density of non-native species in rocky areas therefore could be affected by the relative amount of habitat with appropriate structural complexity.

The Asian shore crab *Hemigrapsus sanguineus* is a relatively recent invader along shorelines of the northwest Atlantic Ocean as well as Europe (Epifanio, 2013). It is most commonly found along rocky shores (Lohrer et al., 2000; Ledesma & O’Connor, 2001) where it has an omnivorous diet and may compete with resident crab species for food and shelter (Jensen, McDonald & Armstrong, 2002; Lohrer & Whitlatch, 2002). This species has reached high densities both in the USA (≥100 individuals m^−2^; Kraemer et al., 2007; O’Connor, 2018) and Europe (up to 70 m^−2^; Dauvin & Dufossé, 2011; Gothland et al., 2013), exceeding those in the native range (<30 m^−2^; Takahashi et al., 1985; Lohrer et al., 2000). However, densities are variable among and within locations both in the USA (O’Connor, 2014; Lord & Williams, 2017) and Europe (Dauvin & Dufossé, 2011; Gothland et al., 2013).

Some of the variability in crab density could be explained by the abundance and arrangement of structural elements (rocks) in different intertidal habitats. For example, in Japan Takada (1999) found that the number of *H. sanguineus* was greater in areas with two compared to one layer of rocks, and Fukui (1988) found that piles of rocks increased local density of crabs. In a study of habitat use in its native range, Lohrer et al. (2000) showed that doubling the density of stones increased *H. sanguineus* density but had no effect on crab size. The present study builds on prior work by standardizing crevice space between rocks, which is difficult to accomplish using natural rocks that vary in size and shape. Artificial shelters of stones attached to concrete pavers in patterns of increasing complexity were deployed in a rocky intertidal area in southeastern Massachusetts, USA, to examine the effect of habitat complexity on crab density. The same shelters were also used in laboratory experiments to assess their influence on crab behavior.

## Methods

### Field experiment

#### Artificial shelter design and construction

Artificial shelters were constructed from gray concrete paver blocks (L = 40 cm, W = 20 cm, H = 5 cm) and quartzite river stones (2–3 cm). Prior to construction, the pavers and stones were rinsed with freshwater and left to air dry for 24 h. River stones were then glued to the pavers using saltwater-resistant Seachem cyanoacrylate Reef Glue™ and left to cure for 24 h. Stones remained glued in place throughout the duration of the experiment.

**Figure 1:**
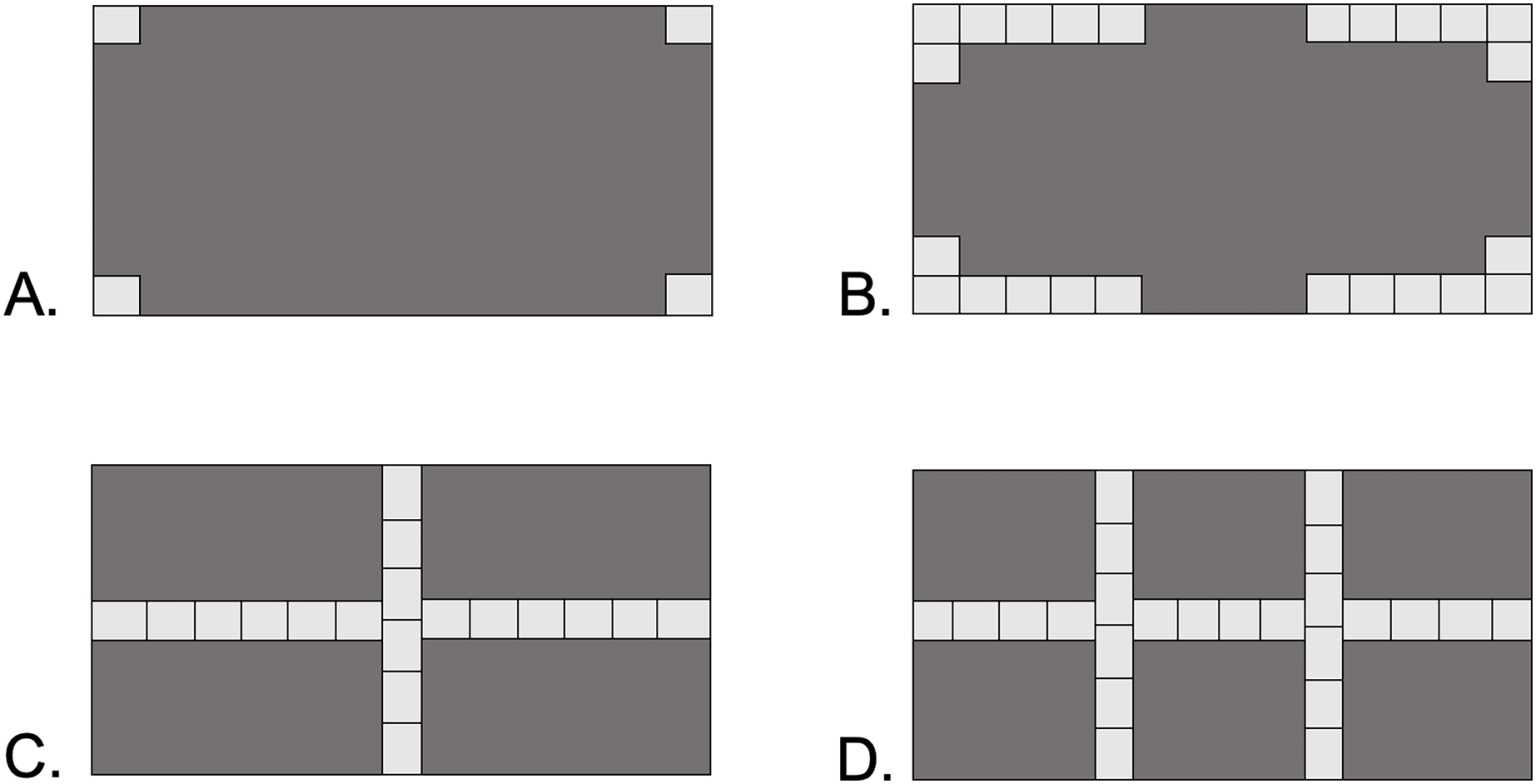
Four paver types used in the field experiment. Dark gray areas show the underside of the 20 × 40 cm paver. Each light gray square represents an individual stone 2–3 cm in size. (A) Open, (B) Corners, (C) Cross, (D) Double-Cross. The area under the pavers without rocks was 650 cm^2^ (B and D), 688 cm^2^ (C), and 775 cm^2^ (A).

Four types of shelter were designed to represent increasing levels of habitat complexity: Open, Corners, Cross, and Double-Cross (Fig. 1). Open pavers had four stones, with a single stone glued to each corner of the paver block. Corner pavers consisted of 24 stones glued along the corners of the paver block, leaving 10 cm gaps in the middle of each perimeter wall. Cross pavers consisted of 18 stones glued in two orthogonal lines running the length and width of the paver block, intersecting at the center-point of the paver. Double-Cross pavers had 24 stones glued in three lines, one down the center length and two perpendicular lines at 13 cm intervals along the width of the paver. The open vertical space under the pavers was 2–3 cm, equivalent to the height of a stone. The surface area of the floor space under each paver type was 650 cm^2^ for Corner and Double-Cross pavers, 688 cm^2^ for Cross pavers, and 775 cm^2^ for the Open pavers.

#### Field experimental procedure

Pavers were deployed in the intertidal zone in Clark’s Cove, New Bedford, Massachusetts (41°35′40.33″N, 70°54′37.45″W) at a tidal elevation of +0.29 m above mean low water, where a pilot study showed crabs were abundant. The intertidal substrate consisted of boulders, cobbles, and pebbles, on a sandy base. Before placing a paver on the substrate, rocks, shells, and other debris were cleared from the site leaving a flat base of sand. Pavers were placed along a transect parallel to the shoreline in a systematic, repeating arrangement of treatments: Open, Corners, Cross, Double-Cross. A blank control paver (*i.e*., no attached river stones) was also placed on each end of the transect in order to remove edge-bias. Pavers were spaced approximately 1 m apart.

All 40 pavers (*n* = 10 per treatment) were deployed in the field for 21 weeks, from 1 June to 28 October 2020. Pavers were surveyed every 7 d in groups of 12–14 over a 3 d period, *i.e*., all pavers were surveyed at 7 d intervals (*n* = 210 for each paver type). During each survey, the pavers were lifted and all crab species present under the paver were collected. Crabs were identified, sexed, and measured for carapace width (CW) and later released several meters away from the pavers. Before replacing the paver, all rocks and debris underneath were removed from the plot.

#### Background population surveys

Natural crab densities at the field site were determined to test how efficiently the pavers attracted *H. sanguineus* from the surrounding intertidal zone. Four surveys were performed at the location of the field experiment. For each survey, three 1 m^2^ quadrats were placed in the intertidal zone, at each end and in the middle of a transect line at the same elevation as the pavers. Rocks, algae, and other debris were cleared from each 1 m^2^ plot, and all crabs present were identified, sexed, measured (CW) and released. Surveys were performed on June 30, August 3, September 16, and November 4, 2020.

#### Statistical analysis of the field experiment

Mean and median crab density for each paver type (Open, Corners, Cross, and Double-Cross) was calculated for each weekly deployment. Log-transformed paver density (*i.e*., natural log (density + 1)) met the assumptions for ANOVA, specifically normal distribution and homogeneity of variances. To test if habitat complexity influenced crab density, a linear mixed model ANCOVA with repeated measures (α = 0.05) was constructed using the function lmer() from the lme4 package in R (Bates et al., 2015). In this model, paver type was a fixed factor, the week surveyed was a continuous covariate, and each paver was a random factor acting as a repeating measure. All four paver types were compared using the function anova() from the car package in R (Fox & Weisberg, 2019). To test for significance of random effects, the repeated measures was assessed using the function ranova() from the package lmerTest in R (Kuznetsova, Brockhoff & Christensen, 2017). A *post-hoc* pairwise comparison was performed to determine any differences in crab densities among paver type using the function emmeans() from the R package emmeans (Lenth, 2022).

A Kolmogorov-Smirnov test was run to determine if the size frequency distribution of crabs in background surveys varied over time using the ks.test() function in the R package dgof. To determine if crabs of particular sizes were taking refuge under the pavers more than in the natural background substrate, four electivity tests were performed comparing crab sizes found under the pavers on dates closest to the background surveys, with bin sizes of 1 mm. Electivity measures the utilization of a resource in relation to its abundance in the environment. Using the recommendations described in Lechowicz (1982), the crab size frequency under the pavers was compared against size frequency in the natural background substrate utilizing Vanderploeg & Scavia’s (1979) relativized electivity index (E*). The index scales from −1 to +1, where −1 indicates avoidance, 0 indicates random selection, and +1 indicates preference. Each crab size was tested for electivity (E*) using the vs_electivity() function from the R package electivity (v.1.0.2, Quintans, 2019). All statistical analyses performed in this study were done using R version 3.5.2 (R Core Team, 2021).

### Laboratory experiment

#### Apparatus design

A 113.6-l acrylic aquarium (L = 91.4 cm, W = 45.7 cm, H = 30.5 cm) with a transparent bottom was suspended 40.6 cm above a flat surface. A Panasonic Lumix DMC-TS30 Digital Camera™ was placed underneath the aquarium, viewing upwards. Given the maximum field of view of the camera, a plexiglass enclosure (L = 54 cm, W = 38 cm, H = 20 cm) was constructed and lined with black waterproof patch and seal tape (H = 10 cm) to create a viewing arena with eight 45° corners (a pilot study showed that crabs would congregate in 90° corners). The overall arena had an interior area of 1,815 cm^2^ with the paver covering 800 cm^2^. The camera’s field of view was divided into a grid of 10 cm × 10 cm squares.

#### Specimen collection and preparation

For each experimental trial, eight adult male *H. sanguineus* were collected near the site of the field experiment. Crabs ranged in size from 14–17 mm CW, large enough to be viewed by the camera, with all claws and legs present. The effective density for each trial was 44 crabs m^−2^. The crabs were placed in a holding tank with running seawater for 24–72 h. Aquarium water was sourced directly from Buzzard’s Bay and kept at ambient temperature and salinity (15 °C–21 °C and 33–34). Air temperature in the lab ranged from 15 °C–17 °C. After collection, the crabs were fed daily with Hikari Tropical Crab Cuisine® pellets and given 10 min to feed, then all uneaten food items were removed. All crabs were starved for at least 24 h prior to the experiment, and no crabs were fed during the experiment.

A total of 1 h to the experiment, all crabs were removed from the aquarium and placed into individual cups where they were left to air-dry for 30 min. When dry, carapace width was measured and each crab was marked with a distinctly colored waterproof nail polish dorsally and ventrally. The crabs were placed back into the cups for another 30 min while the nail polish dried.

#### Laboratory experimental procedure

Using the same four paver types from the field experiment, a single paver was placed in the center of the test arena, with the paver’s longest length running parallel to the longest axis of the aquarium. When viewed through the camera lens, the paver appeared center-frame. The arena border was placed around the paver, set along the edge of the camera’s field of view. One crab was placed into each of the eight 45° corners of the dry arena. The positions of the crabs were recorded for 60 min, with a single image taken every 10 s. The time lapse videos were uploaded from the camera to a MacBook Pro laptop and observed using VLC media player (Version 3.0.6 Vetinari). Every 5 min (every 30 frames), the position of each individual crab relative to the shelter (either under the paver or not) was noted.

At the end of the trial in air, all eight crabs were removed from the arena and placed in individual cups of seawater for 30 min. The arena was filled with seawater and a second trial using the same eight crabs was begun. One crab was placed into each of the eight 45° corners of the arena and its position was recorded as before. Four trials, each using eight crabs, were performed for each paver type. Each crab was used once in aerial and submerged conditions, and then released near the collection site. The laboratory experiments were performed in October and November, 2020.

#### Statistical analysis of the laboratory experiment

To test if pavers accumulated crabs more often when exposed to air or submerged in water, a linear mixed model ANOVA with repeated measures (α = 0.05) was constructed using the function lmer() from the lme4 package in R (Bates et al., 2015). In this model, both paver type and condition (exposed to air or submerged in water) were fixed effects, time was a random effect, and individual crabs were the repeated measure because they were tested in both air and water. The analysis of main effects and interactions was performed using the function anova() from the car package in R (Fox & Weisberg, 2019). To test for the significance of time, a repeated measures was performed using the function ranova() from the package lmerTest in R (Kuznetsova, Brockhoff & Christensen, 2017). To determine significance between condition and paver type, separate *post-hoc* pairwise comparison tests were performed using the function emmeans() from the R package emmeans (Lenth, 2022). Crab activity was also quantified by analyzing the number of positional changes for each crab (*i.e*., the cumulative number of switches in position between under or outside of the paver) over the 60 min period in air and under water. The number of switches was analyzed as a two-way ANOVA with both paver type and condition (exposed to air or submerged in water) as fixed effects. All statistical analyses were done using R version 3.5.2 (R Core Team, 2021).

## Results

### Use of paver types by crabs

A total of 3,485 *H. sanguineus* were collected under pavers across the 21-week field season, along with 19 *Carcinus maenas* and 49 crabs in the family Panopeidae. Asian shore crabs ranged in size from 1.7 to 29.1 mm CW. On 69 occasions (of 840 in total), an entire paver was dislodged or remained underwater and was excluded from analysis.

**Figure 2:**
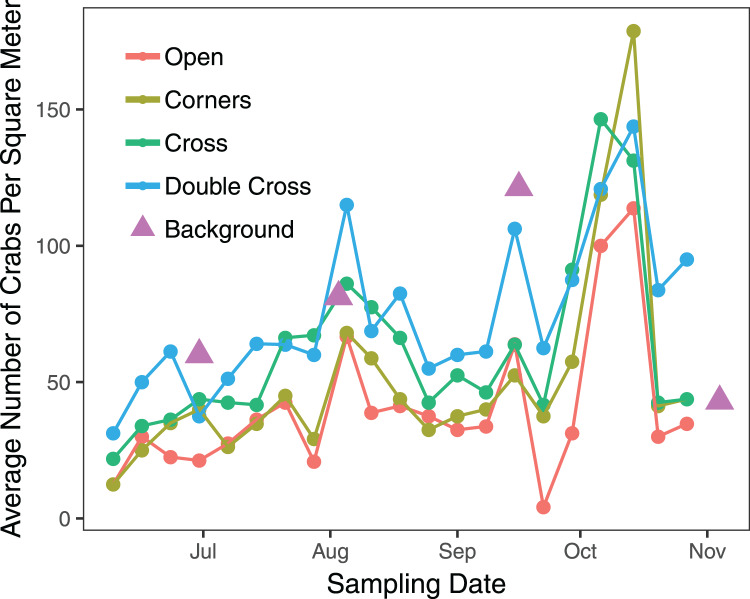
Average weekly density of *H. sanguineus* found under all paver types across the 21-week field season. Triangles show background densities on four dates.

**Figure 3:**
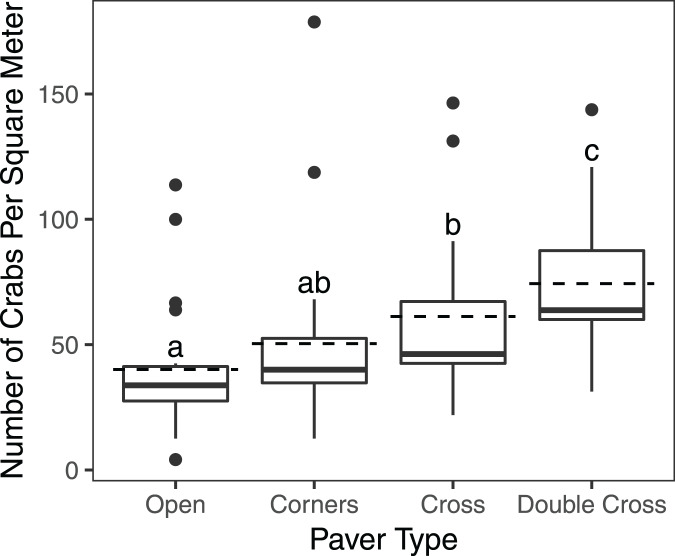
Box and whisker plot showing *H. sanguineus* density under all paver types over 21 weeks. Boxes indicate 25^th^ percentile (Q1), median, and 75^th^ percentile (Q3). Dashed lines indicate mean density. Whisker lines indicate 5^th^ and 95^th^ percentile. Paver types sharing the same letter were not statistically different (*p* < 0.05). Sample sizes were 195 (Open), 192 (Corners), 191 (Cross), and 193 (Double-Cross).

The interaction between treatment and the covariate week was insignificant ( $\chi^2$ = 2.823, df = 3, *n* = 840, *p* = 0.42), but the paver treatment was significant ( $\chi^2$ = 21.1, df = 3, *n* = 840, *p* = 0.0001) (Fig. 2). Double-Cross pavers showed the highest average density of crabs (74.3 crabs m^−2^), while Open pavers showed the lowest average density (40 crabs m^−2^) (Fig. 3). A *post-hoc* pairwise comparison test showed statistical significance between four combinations of paver type: Open and Cross (*p* = 0.0009), Corners and Double-Cross (*p* = 0.0001), Cross and Double-Cross (*p* = 0.0305), and Open and Double-Cross (*p* = <0.0001) (Fig. 3). Across the 21 weekly censuses, the Double-Cross pavers housed the highest average crab density 14 times, whereas the Open pavers housed the fewest crabs in 16 of 21 occasions. Further, the sex ratio of all crabs under the pavers was not significantly different from that in the background surveys ( $\chi^2$ = 2.01, df = 1, *n* = 397, *p* = 0.156).

### Electivity of paver types

**Figure 4:**
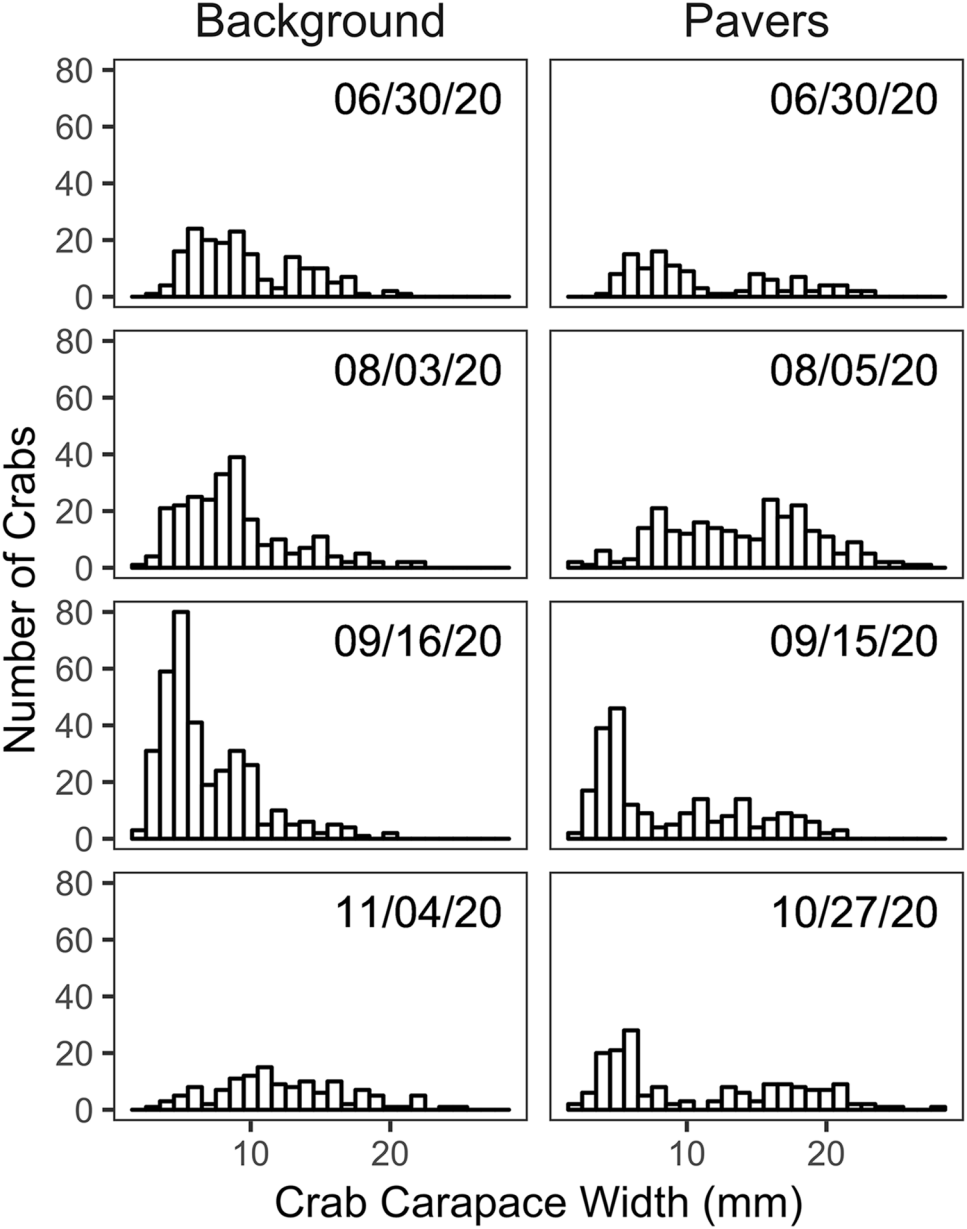
Size frequency histograms of *H. sanguineus* found in background surveys (*n* = 3 on each date, left panel) and under all pavers on similar dates (right panel).

**Figure 5:**
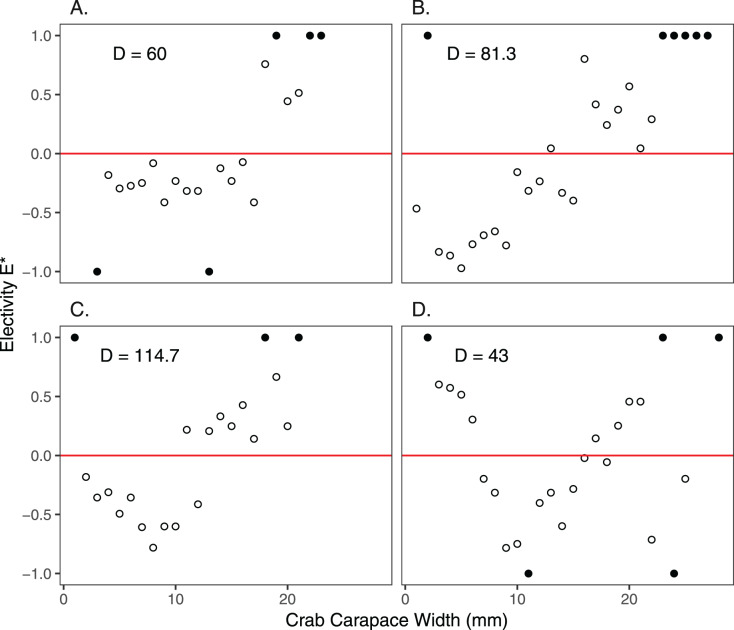
Electivity of *H. sanguineus* for paver shelters *vs* natural substrate based on carapace width (mm). Negative values of electivity indicate avoidance, the red line indicates no preference, and positive values indicate attraction. Comparisons were made on June 30 (A), August 3 and 5 (B), September 15 and 16 (C), and October 27 and November 4 (D). Background crab density (D = #/m^2^) is shown in each panel. Filled circles indicate that crabs of those sizes either were not found in the background survey (values of +1.0) or under the pavers (values of −1.0).

Size frequency distributions of *H. sanguineus* found in the background surveys varied over the duration of the experimental period (*p* << 0.05 for all pairwise comparisons except for June 30 *vs* August 3; Fig. 4). Large crabs were more abundant under pavers than in the natural substrate, *i.e*., showed positive electivity (Fig. 5). In contrast, smaller crabs were less abundant under pavers, especially when background densities were highest in August and September (Figs. 5B and 5C).

### Laboratory experiment results

**Figure 6:**
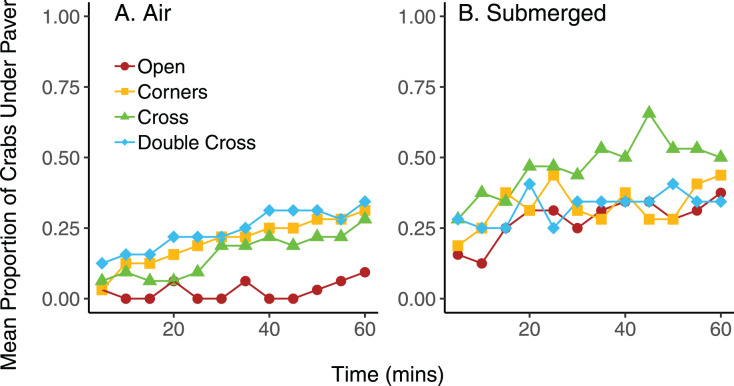
Mean proportion of crabs under the four types of pavers at 5 min intervals over 60 min when exposed to air (A) and when submerged in water (B). Each mean proportion represents an average of four trials, with eight crabs per trial.

**Figure 7:**
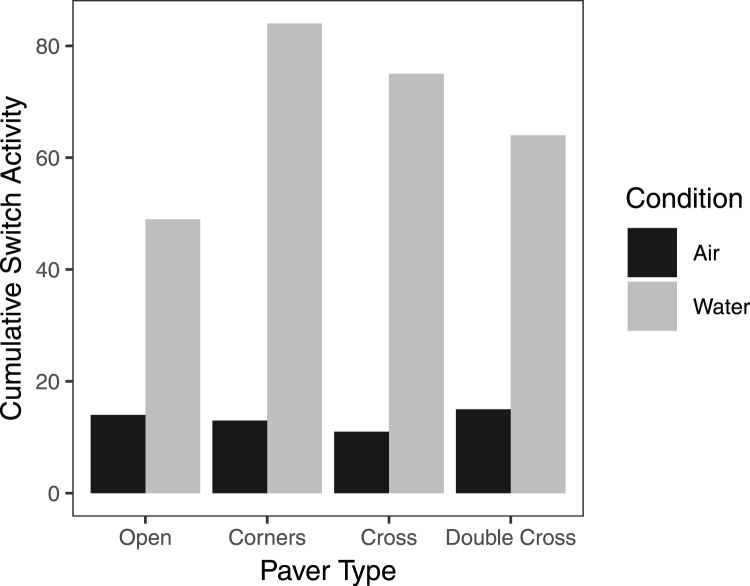
Cumulative switch activity of *H. sanguineus* for all paver types, comparing behavior when exposed to air (black bars), and when submerged in water (gray bars). Bars represent the total number of times crabs moved under or out from under the paver.

On average, pavers tended to accumulate more crabs when they were submerged in seawater, compared to trials exposed to air, regardless of paver type (Fig. 6). However, the interaction between paver type and condition was significant ( $\chi^2$ = 36.9, df = 3, *n* = 16, *p* < 0.001), likely due to the plateauing of paver occupancy in the submerged treatment. Nonetheless, the number of crabs under the pavers increased over the 60 min experimental period for all paver types, whether the crabs were exposed to air or submerged in water. Crabs switched position (under *vs* outside of pavers) more frequently when submerged in water than when exposed to air (F = 6.977, df = 3,24, *p* = 0.0143; Fig. 7). Both paver type and the interaction between paver type and condition were insignificant.

## Discussion

Crab density increased with increasing complexity of the pavers, despite the more complex pavers having less internal surface area. Although the arrangement of stones in the Corners pavers provided a refuge for crabs near the outer edge of the paver, crab density was greater when rocks were arranged in intersecting lines in the Double-Cross pavers, despite the two paver types having equal internal area. This suggests that the interior arrangement of stones in the Double-Cross pavers was more attractive to crabs.

A criticism of studies of this kind is that complexity is often manipulated without controlling for habitable area (Loke & Todd, 2016). While partly true of this study as well, the Corners and Double-Cross pavers had the same internal area but different configurations. The Double-Cross pavers had six separate compartments with eight internal corners whereas the Corners treatment had one large internal space and four corners around the edges. Other studies using natural and artificial habitats have recorded higher invertebrate densities in structurally complex substrates with equal surface area (Warfe, Barmuta & Wotherspoon, 2008; Loke & Todd, 2016).

It is possible but highly unlikely that individual crabs returned to the pavers after being captured and released several meters away. During any paver sampling period, an average of only 190 crabs were caught in total under the pavers, in contrast to an estimated 12,000 crabs in the immediate intertidal vicinity (40 pavers, 1 m apart, in an intertidal zone of approximately 3 m width where crabs were present). In a mark-recapture study, Brousseau et al. (2002) found that crabs released under cement patio pavers very similar in size and composition to ours usually did not return to the same paver and traveled an average of 7.4 m from the paver in 24 h. Moreover, background crab densities in the natural rock habitat were similar to those under the pavers, suggesting that crabs did not preferentially select or avoid pavers in general.

Sizes of crabs occupying shelter spaces under the pavers differed from crab sizes in the natural substrate. Large crabs were found more frequently under the pavers than in nearby rocky substrate, a relationship that increased with background crab density. This suggests that crevice size and shape can limit space for large individuals. Bateman & Fleming (2015) observed preferential selection of larger shelter spaces over small spaces by the crab *Leptograpsus variegatus* when they were threatened and sought shelter on a rocky shore. Habitat refuge space of the appropriate size can affect the abundance of large crabs in a variety of benthic habitats (polychaete reefs: Méndez Casariego, Schwindt & Iribarne, 2004; oyster reefs: Shervette et al., 2004; Margiotta et al., 2016) as well as amphipods occupying macroalgae (Hacker & Steneck, 1990). The difference in paver usage by crabs of different sizes can have implications for crab density as well. Volumetrically, large crabs take up more space than smaller crabs, decreasing the number of crabs that could occupy a given shelter.

Many *H. sanguineus* were observed sharing shelters. This species is more tolerant of the presence of conspecifics than other crab species (Hobbs, Cobb & Thornber, 2017), which is another factor that could contribute to high densities of this invader. While small, newly-recruited juveniles might be subject to cannibalism (Baillie & Grabowski, 2019; Crane & O’Connor, 2021), those that survive and grow to larger sizes can coexist under crowded conditions.

Although increasing paver complexity led to higher crab densities in the field, paver complexity had little effect on accumulation of individuals in the lab. Rather, the simulated tidal condition had a greater effect on crab movement: crabs moved under and out from under the pavers more frequently when they were under water than exposed to air. This result was somewhat unexpected, since crabs leaving shelter should be more vulnerable to aquatic predators. However, crabs might feed more at high tide, and in the natural rocky environment they could move to crevices between rocks for shelter. In contrast, crabs leaving shelter during low tide would be subject to thermal and desiccation stress. This result confirms anecdotal observations of limited movement of *H. sanguineus* during daytime low tides.

The pavers accumulated crabs during the 60 min laboratory trials, especially in air, and number of crabs did not plateau. Moreover, the number of crabs under the pavers in the lab study was lower than expected. The relatively short duration of the experiment (60 min) likely was not long enough for utilization of the pavers by crabs to stabilize. Crabs usually moved either along the perimeter of the arena wall or under the paver during the trial; they infrequently stopped in the middle of the arena space away from the wall or paver. Because the arena wall was black, it might have appeared to the crabs as a potential place of shelter, leading to increased searching behavior along the perimeter.

The Asian shore crab *H. sanguineus* has successfully colonized the shorelines of eastern North America as well as Europe, and achieved high and relatively stable densities in many locations. Areas with the highest crab densities should be impacted the most by this non-native species. If the geographic range of this invader continues to expand, the amount or variability of intertidal habitat complexity may serve as a strong predictor of its density in newly colonized areas. Recent ecological modeling using oceanographic data suggests that the range of *H. sanguineus* could expand substantially in Europe (Karlsson, Obst & Berggren, 2019). If three-dimensional structure is also important (as suggested here), natural resource managers should consider benthic substrate characteristics as well as oceanographic conditions when monitoring the range expansion of this non-native crab.

## Supplemental Information

10.7717/peerj.15161/supp-1 Supplemental Information 1 Crab abundance under shelters in field. The number of crabs under each paver type throughout the experiment.

10.7717/peerj.15161/supp-2 Supplemental Information 2 Crab abundance under pavers in the lab experiment. The number of crabs under pavers in water and in air, for all paver types.

10.7717/peerj.15161/supp-3 Supplemental Information 3 Sizes of crabs in background surveys. The sizes of crabs found in surveys of natural substrate during the field experiment.

10.7717/peerj.15161/supp-4 Supplemental Information 4 Sizes of crabs under pavers in the field. The sizes of crabs under all paver types throughout the field experiment.

## Additional Information and Declarations

### Data Availability

The following information was supplied regarding data availability: The raw measurements are available in the Supplemental Files.
